# Histone H1.5 binds over splice sites in chromatin and regulates alternative splicing

**DOI:** 10.1093/nar/gkz338

**Published:** 2019-05-11

**Authors:** Ohad Glaich, Yodfat Leader, Galit Lev Maor, Gil Ast

**Affiliations:** Department of Human Molecular Genetics and Biochemistry, Sackler Faculty of Medicine, Tel Aviv University, Ramat Aviv 69978, Israel

## Abstract

Chromatin organization and epigenetic markers influence splicing, though the magnitudes of these effects and the mechanisms are largely unknown. Here, we demonstrate that linker histone H1.5 influences mRNA splicing. We observed that linker histone H1.5 binds DNA over splice sites of short exons in human lung fibroblasts (IMR90 cells). We found that association of H1.5 with these splice sites correlated with the level of inclusion of alternatively spliced exons. Exons marked by H1.5 had more RNA polymerase II (RNAP II) stalling near the 3′ splice site than did exons not associated with H1.5. In cells depleted of H1.5, we showed that the inclusion of five exons evaluated decreased and that RNAP II levels over these exons were also reduced. Our findings indicate that H1.5 is involved in regulation of splice site selection and alternative splicing, a function not previously demonstrated for linker histones.

## INTRODUCTION

During splicing, which occurs in the nuclei of eukaryotic cells, introns are removed, and exons are ligated together to form a mature messenger RNA (mRNA). RNA sequences that regulate splicing, such as the 5′ and 3′ splice sites (ss), the poly-pyrimidine tract, the branch site, and splicing regulatory elements, are important for, but are not predictive of all, splicing decisions. It has become apparent that chromatin organization and epigenetic markers also affect splicing (1–8). For example, exons are more occupied by nucleosomes (9–13) and are more CpG methylated (14–16) compared to introns. These features influence splicing due to the coupling of transcription and splicing (17). Transcription is carried out by RNA polymerase II (RNAP II) (18), and splicing occurs while the newly synthesized RNA is still tattered to RNAP II (19). It was shown that RNAP II elongation rate has an effect on exon inclusion levels (20–22) and that RNAP II interacts with several splicing factors including the spliceosomal component U1 snRNP (23–26). Thus, splicing can be probably regulated by chromatin and epigenetic markers through kinetic coupling (6).

Chromatin organization in eukaryotes is achieved mainly by the double-loop wrapping of DNA around two sets of four core histone proteins H2A, H2B, H3, and H4 that together form a core nucleosome (27). Higher order chromatin organization results from the binding of a fifth histone, the linker histone H1, at the DNA entry and exit sites near the nucleosome dyad (27). Linker histones are a heterogeneous family with higher sequence variability between species compared to core histones. In mammals, eleven H1 subtypes have been identified: five somatic H1 variants (H1.1 to H1.5), the replacement H1 (H1.0), germ cell-specific H1s (H1t, H1T2, HILS1 and H1oo) and H1x (28–30).

Although it is known that core histones are preferentially located over exons (9–13), a possible role for the linker histone in splicing has not been tested to date. Moreover, since throughout evolution exons have maintained a length that coincides with the length of DNA around mono-nucleosome (∼146 base pairs (bp)) (9,31), we hypothesized that linker histones bind to linker DNA encoding splice sites. H1 might slow the elongation rate of RNAP II as the enzyme tries to overcome the nucleosomal barrier, which in turn can promote exon inclusion via kinetic coupling with the splicing process. In support to this notion, it was found that linker histones associate with RNAP II (32,33) and with several splicing factors (34). In addition, H1 also prevents nucleosome sliding (35,36), and thus can maintain the core histone preferential location over exons. It is also possible that linker histone is actually the primary chromatin component that aids exon recognition rather than core histones.

There is evidence that linker histone variants have different functions. For example, knockout and knockdown studies of single variants or a few together have shown that expression of a small but specific subsets of genes is affected by each variant (37–41). H1x and H1.0 have different expression patterns than H1.1–H1.5 (42,43), and H1.1 has a binding profile different from those of H1.2–H1.5 (44). H1.5 accumulates in differentiated human lung fibroblasts but not in undifferentiated human embryonic stem cells (45), and H1.0 levels are reduced in tumor cells that exhibit long-term self-renewal ability compared to those with less self-renewal capacity (46). Linker histones interact with many different proteins, mostly through their unstructured C-terminal domains. For instance, linker histone variant H1.0 can bind more than thirty different splicing factors (34), and H1.4 selectively binds HP1 (47), a known chromatin organizer that influences splicing (48). Therefore, it is also possible that specific H1 variants regulate specific splicing events.

Here, we analyzed a ChIP-seq dataset of linker histone variant H1.5 in human lung fibroblast IMR90 cells and RNA-seq datasets from wild-type and H1.5-deficient IMR90 cells (45). H1.5 is the longest in length (226 amino acids) of the seven somatic subtypes (H1.1 to H1.5, H1.0 and H1X) (28). H1.5 possess a relatively long C-terminal domain (CTD) consisting of 114 amino acids; of these, 46 are lysine. Thus, the H1.5 CTD has more positively charged amino acids than the other somatic linker histones (28). The length of the CTD partially correlates with the affinity of linker histones for chromatin (49), and H1.5 has a longer residence time in chromatin than the other linker histone subtypes (28). We found that H1.5 binds chromatin encoding splice sites of short exons and assists in their inclusion. Further, we found that H1.5 promotes RNAP II pausing at 3′ splice sites (3′ss), which probably increases the likelihood that an exon will be detected by the splicing machinery. Last, we found that H1.5 binding near splice sites occurs in genes that are highly alternative spliced and that encode proteins with binding ability.

## MATERIALS AND METHODS

### Cell culture, transfection and RNA extraction

IMR90 cells, a human primary lung embryo fibroblast line, were cultured in EMEM (ATCC) containing 10% FBS. At 70% confluence, cells in wells of six-well plates were transfected with 25 nM siRNA targeting *H1.5* (Dharmacon, MU-012049–00) or with the control siGENOME non-targeting human siRNA pool #2 (Dharmacon) using Lipofectamine RNAiMAX transfection reagent (Invitrogen). Cells were harvested after 48 h, and RNA was extracted using TRI reagent (Sigma-Aldrich). cDNA was synthesized with RT-FLEX (Quanta). qPCR was performed using KAPA SYBR FAST Universal qPCR kit (KAPA Biosystems) according to the manufacturer's instructions using different exon-exon junction primer pairs to amplify the inclusion isoform. All qPCR reactions were run on ABI step-one-plus thermocycler. Primer sequences will be provided upon request. In Figure 7D, expression levels were measured following H1.5 siRNA or scramble siRNA for constitutive exons in the five selected genes.

### MNase treatment

Nuclei were suspended in MNase digestion buffer (0.32 M sucrose, 50 mM Tris–HCl, pH 7.5, 4 mM MgCl_2_, 1 mM CaCl_2_) supplemented with 0.1 mM PMSF. MNase (10 U/10^6^ nuclei, Worthington) was added, and samples were incubated at 37 °C for 10 min. The reaction was stopped by the addition of 20 mM EDTA.

### ChIP assay

Approximately 3 million cells per sample (treated with siH1.5 or control siRNA) were crosslinked for 10 min in 1% formaldehyde. Crosslinking was quenched by the addition of 125 mM glycine. Cells were washed twice with PBS, pellets were subjected to nuclei extraction, and chromatin was fragmented by MNase and sonication to 100–500 bp. Immunoprecipitation using an antibody to RNA polymerase II (Abcam, ab76123) was performed. For each immunoprecipitation reaction, 40 μl of protein-A and G Dynabeads (Invitrogen), pre-incubated with 3 μg of the antibody were added, and samples were incubated for 16 h at 4 °C. The beads were washed six times with RIPA buffer (0.1% deoxycholate, 0.1% SDS, 1% Triton X-100, 10 mM Tris–HCl, pH 8.1, 1 mM EDTA, 140 mM NaCl), twice with RIPA-high salt buffer (0.1% deoxycholate, 0.1% SDS, 1% Triton X-100, 10 mM Tris–HCl, pH 8.1, 1 mM EDTA, 360 mM NaCl), twice with LiCl wash buffer (250 mM LiCl, 0.5% NP-40, 0.5% deoxycholate, 1 mM EDTA, 10 mM Tris–HCl, pH 8.1), and twice with TE buffer (10 mM Tris–HCl, pH 8.1, 1 mM EDTA). DNA was eluted from the beads with Elution buffer (0.5% SDS, 300 mM NaCl, 5 mM EDTA, 10 mM Tris–HCl, pH 8.1) and incubated for 30 min in a thermo-shaker at 65 °C. RNase A (Sigma, 1 μl of 10 mg/ml stock) was added to the eluted samples. After incubation for 30 min at 37 °C, 1.5 μl Proteinase K (NEB) was added, and samples were incubated for 16 h at 65 °C. DNA was purified using phenol extraction. qPCR was performed using KAPA SYBR FAST Universal qPCR kit (KAPA Biosystems). qPCR reactions were run on a ABI step-one-plus thermocycler. The sequences of qPCR primers used will be provided upon request.

### Gene annotations

**Figure 1.**
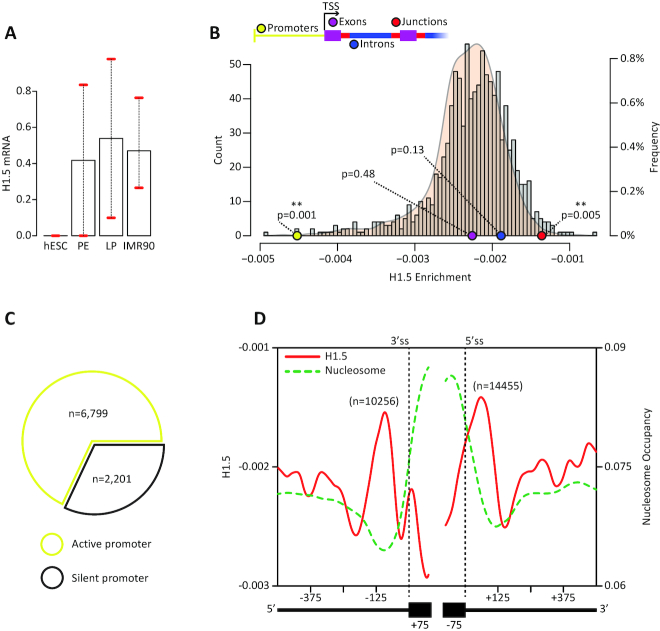
H1.5 binds DNA over splice sites. (**A**) *H1.5* mRNA FPKM values from mRNA-seq for human embryonic stem cell, primitive endoderm, lung precursor, and human lung fibroblasts. Plotted are means with a confidence interval. (**B**) Histogram (gray bars) and density plot (brown area) show the distribution of H1.5 mean occupancy of 1000 random samples. Each sample contained 10 000 random intervals of 50 bp from gene regions. The left axis denotes the histogram counts and the right axis denotes the density plot frequencies. Colored dots represent the means of H1.5 occupancies of particular gene regions. The *P* values are based on a randomization test. (**C**) Pie chart shows the proportions of active and silent promoters. (**D**) H1.5 occupancy and nucleosome occupancy plotted across exon-intron junctions of 179 452 internal exons at single-base resolution. The figure shows values for 75 bp of the exon and 500 bp of the intron at both junctions.

The RefSeq *refGene* table was downloaded from the UCSC table browser (https://genome.ucsc.edu/cgi-bin/hgTables) in GTF format. Lines with scaffold or mitochondrial chromosomes or with features other than exons were removed. Gene description for human RefSeq accessions were downloaded from (ftp://ftp.ncbi.nih.gov/refseq/H_sapiens/mRNA_Prot/) and added to the table. Genes with a gene description other than ‘mRNA’ or ‘long non-coding RNA’ or ‘non-coding RNA’ were also removed. Using in-house Perl scripts, coordinates for introns were added. In addition, duplicated or overlapping exons were removed, leaving only the exon annotated in most transcripts, regardless of strand position. The position of each exon in a transcript was added to the table, and the first and last exons in each transcript were taken out into separated lists leaving only the internal exons. Overall, our table consisted of 179 452 internal exons. In Figure 1B, duplicated or overlapping regions were removed using mergeBed v2.17.0 (50), and the *junctions* category (first and last 100 bp of each intron) was constructed using an in-house Perl script. For Figure 1B, we evaluated 28 235 promoters, 181 009 internal exons, 149 940 introns, and 299 880 junctions.

### Datasets

We downloaded raw reads from the Gene Expression Omnibus (https://www.ncbi.nlm.nih.gov/geo/) for H1.5 ChIP-seq in IMR90 cells (GSE26979), RNA sequencing data of wild-type IMR90 cells and of H1.5-deficient IMR90 cells (GSE41761), data on nucleosome positioning in IMR90 cells (GSE44985), raw RNAP II ChIP-seq data in IMR90 cells (GSM1055822), and H3K4me3 ChIP-seq in IMR90 cells (GSE43070). We downloaded processed data of DNase I hypersensitive sites sequencing in IMR90 cells (GSM1008586). The CpG methylation profile in IMR90 cells obtained using bisulfite-seq was done by Ziller *et al.* (51) (GSM1204464). We used the table that lists the numbers of methylated reads/the total reads and the percent of methylated reads for all observed CpGs. We downloaded RNA sequencing data of human embryonic stem cells (GSM1694954), primitive endoderm (GSM3494684), and lung precursor (GSM2893715).

### Alignment to the human genome

Sequencing reads were aligned to the human genome (Assembly hg19, GRCh37 Genome Reference Consortium Human Reference 37) using Bowtie2 v2.1.0 (52) for reads derived from DNA fragments and TopHat v2.0.9 (53) for reads derived from mRNA fragments. Bowtie2 was run with default parameters and TopHat was run with default parameters and fed with RefSeq genes annotation. To improve read quality, we trimmed H1.5 ChIP-seq and input reads, leaving the first 51 bp, and we trimmed the wild-type and the H1.5-knockdown RNA-seq reads, leaving 57 bp from base 4 to 60. PCR duplicated reads and low mapping quality reads were filtered out using samtools rmdup and samtools –q 30 functions, respectively.

### ChIP-seq and MNase-seq occupancy

The UCSC bigWig format was chosen to represent the sequencing coverage and depth. Sequencing depth files at single-base resolution were created using the tool bam2wig.pl (http://search.cpan.org/∼tjparnell/Bio-ToolBox-1.44/) and for each base a normalized reads-per-million value was calculated and assigned considering all the reads that span that base. In order to better represent the protein location in ChIP-seq experiments, prior to the depth calculation aligned reads from the ChIP-seq library, but not from the input library, were shifted several bp toward the speculated middle of their originating DNA fragment, estimated by the level of sonication in the library preparation stage. We shifted H1.5 ChIP-seq reads 87 bp downstream on the read strand to account for a middle of a mean fragment size of 225 bp. We shifted RNAPl II and H3K4me3 ChIP-seq reads 127 bp to account for a mean fragment size of 290 bp. Last, the normalized input coverage was subtracted from the normalized ChIP-seq coverage to represent the difference between the two samples and the final occupancy value for each base. For MNase-seq, aligned reads were extended to the length of 147 bp prior to the coverage and depth calculation. We also constructed a binary bigWig file to represent the GC content in the hg19 human genome assembly using in-house Perl scripts.

### Data extraction and clustering

Data stored in bigWig format was extracted using bwtool (54) fed with relevant BED files. bwtool was also used to align feature to their start or end coordinate and to calculate the mean single-base values for multiple features in a given BED file. bwtool was also applied to perform k-means clustering for multiple features in a given BED file.

### Gene expression and isoform abundance calculation

RNA quantification was performed using Cufflinks v2.2.1 (55) fed with our RefSeq genes table and with default parameters and a minimum value of 0.1 fragments-per-kilo-million (fpkm) was set for a fragment to be considered as expressed. Differential expression was evaluated with Cuffdiff v2.2.1. Percentage-spliced-in (PSI) for alternative exons and for alternative 3′ss and alternative 5′ splice site (5′ss) isoforms were estimated with MISO v0.4.9 (56). We estimated PSI for all expressed internal exons in our modified RefSeq exons table, converted into GFF3 format by in-house Perl script, and with default parameters. MISO makes use of informative reads that span the tested exons and/or its flanking exons. Exons with no informative reads were discarded from relevant analyses. Exons with at least one informative read that supported their skipping event were labeled as alternative. The rest of the exons were labeled as constitutive. We analyzed the RefSeq genes table with ASTALAVISTA (57) to identify exons with alternative 3′ss or 5′ss, and we used MISO to estimate the PSI of each alternative event. Differential PSI between wild-type and H1.5-deficient conditions was calculated by MISO. We filtered significant events with the parameters –num-inc 1 –num-exc 1 –delta-psi 0.1 –num-total 10 –bayes-factor 1.

### Splice-site strength

The algorithm to calculate the strengths of donor and acceptor sequences is based on the program described by Shapiro et al. (58). The tables used to calculate the splice site strength are based on the analysis performed by Carmel *et al.* of 45 552 splice sites (59). We applied those tables within the algorithm to obtain 5′ss and 3′ss strength for each exon in our RefSeq exons table using an in-house Perl script.

### Statistical analysis

Statistical analysis was done using R (version 3.2.1). Significance level was set to 0.05. A single asterisk denotes *P* < 0.05 and double asterisks denotes *P* < 0.01.

## RESULTS

### H1.5 binds DNA over splice sites

In order to examine the role of histone H1.5 in splicing regulation, we focused on a possible role in human lung fibroblasts (IMR90) cells. Levels of H1.5 are higher in human lung fibroblasts and other differentiated cells than in early lineage stage cells (45). We measured *H1.5* mature mRNA levels in human embryonic stem cells (hESC), primitive endoderm, lung precursor, and IMR90 cells. hESCs had no detectable *H1.5* mRNA, and its levels were indeed elevated throughout lung differentiation (Figure 1A).

Next, we tested H1.5 occupancy over genes in the chromatin of IMR90 cells. We applied a randomization test. Genes were defined as the locus starting 2000 bp upstream of a transcription start site and ending at the transcription termination site. We selected 10 000 random intervals of 50 bp within the areas we defined as genes, and computed the overall H1.5 mean occupancy of the sample. We repeated this step 1,000 times to form a baseline. To test H1.5 occupancy over exons, introns, promoters, and junctions, we selected 10 000 random intervals of 50 bp within each category and computed the overall mean H1.5 occupancy as we did previously (Figure 1B). Junctions were defined as the first and last 100 bp of each intron, and those regions were removed from the intron category. H1.5 mean occupancy over junctions was higher than the baseline (*P* = 0.005), and H1.5 mean occupancy over promoters was lower than the baseline (*P* = 0.001). Promoters were previously shown to be mostly depleted of H1.5 in other cells (60), probably to maintain an open chromatin state that can allow transcription. To confirm this assumption, we measured the expression of genes in the promoters set, and found that 68% of them are active (Figure 1C).

To validate that H1.5 binds over splice sites, we plotted the mean H1.5 occupancy of 179 452 internal exons (see Methods) at single-base resolution across 100 bp of exon and 500 bp of intron sequences around each splice site (Figure 1D; left axis). H1.5 signal peaked on introns close to the exon–intron junctions. To better characterize this binding, we evaluated mean nucleosome occupancy over these regions (Figure 1D; right axis). We observed, as expected, that core histones mark the location of the exon sequences between the adjacent splice sites. This nucleosome-linker histone structure suggests that H1.5 might be involved in splice site detection.

As the overall mean of H1.5 occupancy was relatively low, we wanted to focus on the subset of exons with high H1.5 occupancy over their splice sites. Hence, we applied k-means clustering to group exons into arbitrary five groups based on the H1.5 signal over exon–intron borders; one clustering was created for the 3′ss (Supplementary Figure S1A) and another for the 5′ss (Supplementary Figure S1B). There were two subsets of exons that exhibited H1.5 binding near the 3′ss (blue) or near the 5′ss (green) but not at both sites simultaneously. We intersected the exons that exhibited H1.5 binding near 3′ss in both clustering runs and intersected the exons that exhibited H1.5 binding near 5′ss in both clustering runs (Supplementary Figure S1C). These intersections were statistically significant (hypergeometric test; *P* < 2.2 × 10^−16^). We identified 10,256 exons with H1.5 binding on the 3′ss and 14,455 exons with H1.5 binding on the 5′ss. In addition, we intersected the exons that had H1.5 bound at a distant location, several hundred nucleotides away from splice sites, in both clustering runs (Supplementary Figure S1C; purple and yellow). These intersections were also statistically significant (hypergeometric test; *P* < 2.2 × 10^−16^). We identified 13 853 exons with distant H1.5 binding relative to the 3′ss and 18 241 exons with distant H1.5 binding relative the 5′ss. Overall we identified 24 711 H1.5-marked exons and 32,094 H1.5-distant exons among a population of 179 452 internal exons; the remaining 122,647 exons we defined as unmarked.

### H1.5 binding near short exons correlates with inclusion levels and RNAP II pausing

**Figure 2.**
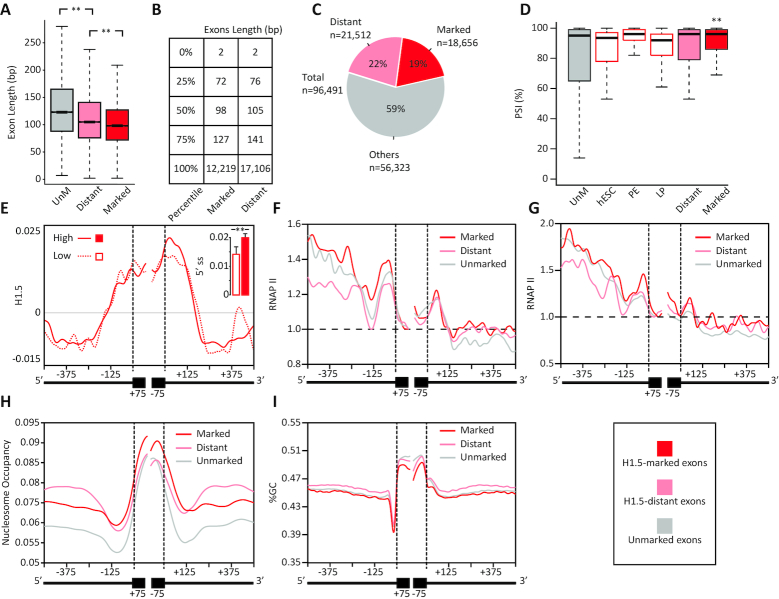
H1.5 binding near short exons correlates with inclusion levels and RNAP II pausing. (**A**) H1.5-marked exons are significantly shorter than unmarked exons. The boxplots show the lengths of H1.5-marked (*n* = 24 711), H1.5-distant (*n* = 32 094), and unmarked (*n* = 24 097) by H1.5; *P* < 0.01, *t*-tests. (**B**) Exon length quartiles for H1.5-marked and H1.5-distant exons. (**C**) Pie chart of the population of exons 127 bp or less in length with H1.5-marked (red) and H1.5-distant (pink) proportions shown. (**D**) H1.5-marked exons are more often included in mRNA than are unmarked exons. Mean PSIs of alternative exons, 80 to 120 bp long, within the H1.5-marked (*n* = 430), H1.5-distant (*n* = 479), hESC (*n* = 338), primitive endoderm (*n* = 342), lung progenitors (*n* = 294), and unmarked groups (*n* = 333) are shown (± SEM); *P* < 0.01, Wilcoxon rank sum test. (**E**) Plot of H1.5 mean signal for alternative exons with PSI < 30% (*n* = 185) and with PSI > 70% (*n* = 752) over a 575-bp window around splice sites. The inset plot shows the mean signal of the first intronic 100 bp of the 5′ss; *P* < 0.01, Wilcoxon rank sum test. (**F**) Mean RNAP II occupancy around H1.5-marked, H1.5-distant, and H1.5-unmarked exons at single-base resolution across 3′ss and 5′ss regions with a 575-bp window around each splice site. The signals were normalized to the value of the first base of the exon. (**G**) Mean RNAP II occupancy around alternatively spliced H1.5-marked, H1.5-distant, and H1.5-unmarked exons across 3′ss and 5′ss regions with a 575-bp window around each splice site. The signals were normalized to the lowest value of each signal. (**H**) Nucleosome occupancy for H1.5-marked, H1.5-distant, and unmarked exons across 3′ss and 5′ss regions with a 575-bp window around each splice site. (**I**) GC content for H1.5-marked, H1.5-distant, and unmarked exons across 3′ss and 5′ss regions with a 575-bp window around each splice site.

We computed the mean length of H1.5-marked and H1.5-distant exons and compared them to the mean length of 24,701 unmarked internal exons randomly selected from the same genes that contained H1.5-marked exons (Figure 2A). H1.5-marked and H1.5-distant exons were significantly shorter than the control group of unmarked exons (*P* < 0.01, two sample t-test). In addition, H1.5-marked exons were shorter than H1.5-distant exons: 75% of the H1.5-marked exons were 127 bp or less, whereas 75% of the H1.5-distant exons were 141 bp or less (Figure 2B). Overall, H1.5-marked exons and H1.5-distant account for 19% and 22%, respectively, of 96 491 short internal exons (up to 127 bp) present in the total population (Figure 2C). We also observed that H1.5-marked exons, but not H1.5-distant exons, had slightly stronger splice sites (see Methods) than unmarked exons (Supplementary Figure S2A and B). This result implies that H1.5 might favor specific sequence composition of linker DNA. In order to test if H1.5 binding to splice site regions is linked to exon inclusion in mature mRNA, we estimated the percentage spliced in (PSI; see Methods) of alternative exons in IMR90, hESC, primitive endoderm, and lung precursor cells. We compared the inclusion levels of H1.5-marked, H1.5-distant, and unmarked exons 80–120 bp long that are alternatively spliced. In addition, we also measured the inclusion levels of the 80–120 bp H1.5-marked alternative exons set in hESC, primitive endoderm, and lung precursors. In IMR90 and primitive endoderm, H1.5-marked exons had the highest PSIs values (Figure 2D), suggesting that H1.5 binding to DNA encoding splice sites may facilitate exon inclusion. To further validate this result we plotted the H1.5 signal around alternative exons that were included at low frequency (PSI < 30%) and at high frequency (PSI > 70%) in IMR90 cells (Figure 2E). Highly included alternatively spliced exons had higher H1.5 signal over the 5′ss regions than exons included at low frequency, and the difference was statistically significant (Figure 2E, *P* < 0.01, Wilcoxon rank sum test).

Next, we tested for RNAP II presence around H1.5-marked and unmarked exons using previously published ChIP-seq data of RNAP II in IMR90 cells (44). We normalized the signals to the lowest value of each signal in order to account for different expression levels between groups. We found that RNAP II stalls near the 3′ss and that this stalling was more frequently observed on H1.5-marked exons than unmarked exons and H1.5-distant exons (Figure 2F). We also plotted RNAP II signal around alternatively spliced H1.5-marked, H1.5-distant, and unmarked exons and observed similar results (Figure 2G). This implies that H1.5 associated with an ‘exonic’ nucleosome, a nucleosome that is wrapped by an exon and splice sites, slows RNAP II processivity leading to enhanced exon inclusion (Figure 2D).

We also measured nucleosome occupancy for H1.5-marked, H1.5-distant, and unmarked exons in IMR90 cells (Figure 2H). H1.5-marked exons had the highest nucleosome occupancy level, and unmarked exons had the lowest level. Thus, nucleosome occupancy is correlated with H1.5 binding. In addition, we calculated frequencies of guanine-cytosine dinucleotides (GpC), and, interestingly, we found no significant differences among the groups of exons (Figure 2I). Cytosine nucleotides are often methylated, and exons and introns have different DNA CpG methylation levels (14).Therefore, we analyzed the correlation of this epigenetic mark with H1.5 binding. We calculated the proportion of CpG methylation around H1.5-marked, H1.5-distant, and unmarked exons (Supplementary Figure S2C). We observed a higher proportion of DNA CpG methylation around the H1.5-marked and H1.5-distant exons compared to the control group of unmarked exons. We also plotted the mean proportion of methylated CpGs over the exons included at high and low frequencies from both the H1.5-marked, H1.5-distant, and unmarked exons groups (Supplementary Figure S2D). Again, levels of methylation were higher around alternatively spliced H1.5-marked and H1.5-distant exons than around the unmarked exons. Overall, these findings suggest that H1.5 and DNA CpG methylation are correlated. It is likely that DNA methylation enhances H1.5 binding; however, it is possible that H1.5 promotes DNA methylation, as linker histones have been shown to bind certain DNA methyltransferases (61). The difference in the methylation levels over the upstream intron also raises the possibility that splicing contributes to methylation maintenance.

Next, we analysed the function of the genes hosting H1.5-marked and H1.5-distant exons (Supplementary Figure S2E and F). We randomly selected 3000 genes of 11 232 genes with H1.5-marked exons and 3000 genes from 12 562 genes with H1.5-distant exons for functional annotation using DAVID (62). The top ranking term for both H1.5-marked and H1.5-distant genes was alternative splicing, which means that these genes can produce more than a single isoform. In addition, H1.5-marked genes were enriched for terms nucleotide-binding, metal-binding, and beta-transducin (WD) repeat.

### H1.5 distance from a splice site affects exon inclusion.

**Figure 3.**
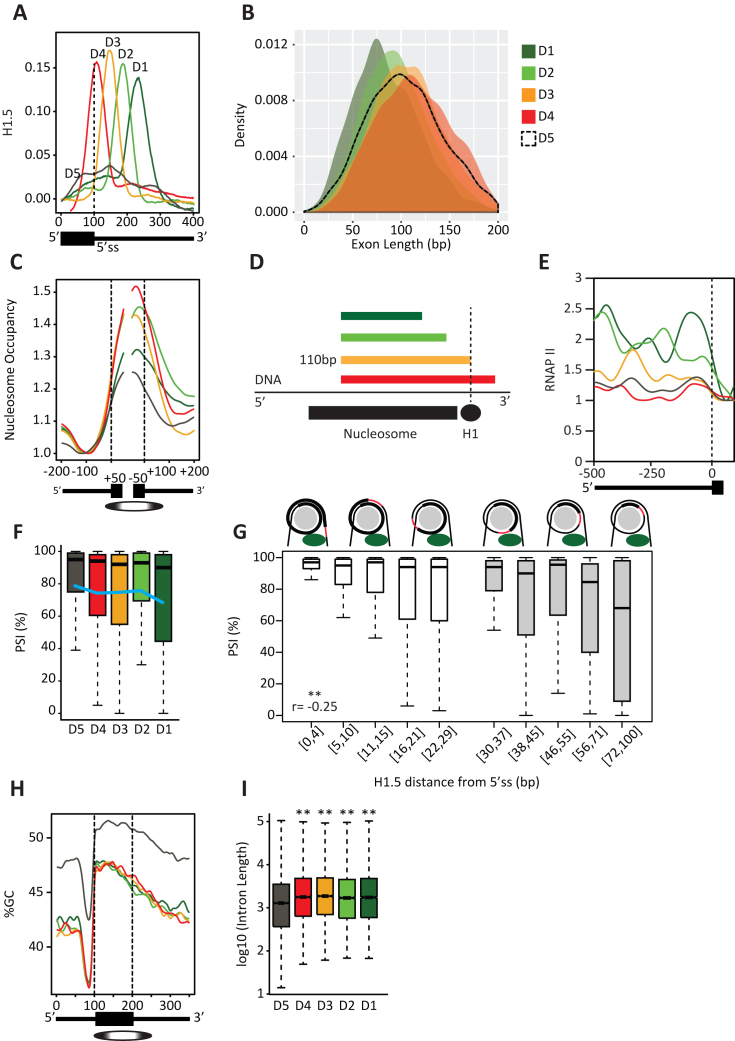
H1.5 distance from a splice site affects exon inclusion. (**A**) The exons marked by H1.5 at their 5′ss (*n* = 14 455) were aligned at 5′ss junction and clustered into five groups (D1–D5) based on H1.5 mean occupancies across 100 bp of exon sequence and 300 bp of intron sequence at single-base resolution. (**B**) Exon length (bp) distribution for each of the five groups D1–D5; (**C**) Mean nucleosome occupancy at single-base resolution for each of the groups D1–D5 across 200 bp of intron and 50 bp of exon. Signals were normalized to the lowest value for each individual cluster. Ellipse denotes the likely nucleosome location. (**D**) Drawing of the absolute distance between the 5′ss and H1.5. (**E**) Mean RNAP II occupancy for each of the five groups D1–D5. The signals were normalized to the lowest value of each signal. (**F**) Percentage of alternative exon inclusion within each group D1–D5. (**G**) Percentage of alternative exon inclusion relative to the absolute distance of H1.5 from a splice site. Each box presents a different distance. The drawing shows the distance between H1 (green) and the splice site (red); *P* < 0.01, *r* = −0.25, Pearson's product-moment correlation (**H**) Mean GC content at single-base resolution across a 350-bp region around the exons in groups D1–D5 with the exons aligned at start points. Ellipse marks the likely nucleosome location. (**I**) Flanking intron length (bp) distribution for each of the groups D1–D5 **P < 0.01, Wilcoxon rank sum test.

To observe the binding of H1.5 at higher resolution, we clustered H1.5-marked exon-intron junctions into arbitrary five groups. We clustered the signals twice, one run around the 5′ss (Figure 3A; groups D1 *n* = 1,972; D2 *n* = 2416; D3 *n* = 2419; D4 *n* = 2417; D5 *n* = 5231) and a second run around the 3′ss (Supplementary Figure S3A; groups A1 *n* = 1495; A2 *n* = 1697; A3 *n* = 1736; A4 *n* = 1670; A5 *n* = 3658). In some exons, H1.5 bound close to the splice site, whereas in others it was up to 120 bp away (Figure 3A and Supplementary Figure S3A). Strikingly, the distance between the location of the H1.5 signal from the 5′ss negatively correlated with exon length (Figure 3B). The same correlation was observed for the 3′ss (Supplementary Figure S3B).

The position of H1.5 near the splice site of short exons must be influenced by the location of the hosting nucleosome. Therefore, we plotted the mean nucleosome occupancy signal for the clustered groups of exons; we normalized each individual signal to its own lowest value in the plot. (Figure 3C and Supplementary Figure S3C). We observed that when H1.5 was bound at the 5′ss, nucleosomes were aligned to the exon start point near the 3′ss. This nucleosome positioning probably directs histone H1.5 toward the end of the exon. In contrast, when H1.5 was bound to the 3′ss, nucleosomes were aligned to the exon end near the 5′ss directing H1.5 to bind near the start of the exon (Supplementary Figure S3C). Since the length of the DNA around the nucleosome (∼146 bp) is typically longer than the length of H1.5-marked exons (Figure 2A) and since nucleosomes are aligned to either the start or end of an exon, one of the splice sites sequences will be bound by core histones. Hence, the nucleosome position explains our earlier observation of a negative correlation between the exon length and the distance of H1.5 from a splice site (Figure 3D and Supplementary Figure S3D). We also examined RNAP II occupancy over groups D1 to D5 and A1 to A5 and found that D5 and A5 had decreased RNAP II pausing near the exon start compared to the other groups (Figure 3E and Supplementary Figure S3E), implying that H1.5 on a well-positioned nucleosome interferes with RNAP II progress.

Next, we estimated the inclusion of alternatively spliced exons within each group. At the 5′ss, for groups D1–D4, there was a positive correlation between the position of H1.5 from a splice site and the inclusion level of the alternatively spliced exon (Figure 3F). At the 3′ss, we observed a similar trend (Supplementary Figure S3F). We also attempted to measure inclusion levels relative to absolute distance between splice sites and H1.5. Based on the nucleosome signal in Figure 3C, we estimated that nucleosomes are aligned ∼35 bp upstream of the first base of the exon, and based on the nucleosome signal in supplementary Figure S3C, we estimated that nucleosomes are aligned roughly 45 bp downstream of the last base of the exon. Since nucleosomes are wrapped by approximately 146 bp of DNA (31), we calculated that 110 bp is the optimal exon length for its 5′ss to be occupied by H1.5 and that the optimal length for an exon with H1.5 at the 3′ss is 100 bp. We observed a negative correlation between the distance of H1.5 from a splice site and level of exon inclusion at both 5′ss and 3′ss (Figure 3G and Supplementary Figure S3G). Strikingly, we observed this correlation over two ranges of distances; a range between 0 to ∼30 bp (Figure 3G and Supplementary Figure S3G; white boxes) and a range between ∼31 to 100 bp (Figure 3G and Supplementary Figure S3G; gray boxes). Considering that DNA circles the nucleosome twice, we suggest that if the length of an exon is roughly 75 bp, the 5′ss or the 3′ss are in contact with H1.5.

The nucleosome location is affected by GC content (63). Therefore, we also measured the GC content level for each group of exons. We aligned the exons in groups D1 to D5 to their start point (Figure 3H), and we aligned the exons in groups A1 to A5 to their end point (Supplementary Figure S3H). We observed that GC content increased dramatically at the aligned borders in concordance to the elevated nucleosome occupancy signal observed. This evidence suggests that GC content architecture directs nucleosome positioning and that nucleosome positioning dictates the location of histone H1.5 binding.

The clustering algorithm also identified a fifth group of exons with low H1.5 occupancy (Figure 3A, group D5; Supplementary Figure S3A, group A5). Interestingly, these two groups had the lowest nucleosome signals when compared to the other groups at the same splice site, suggesting that there is no (or only weak) nucleosome alignment to direct H1.5 binding. We also observed that groups D5 and A5 had higher GC content than groups D1–D4 and A1–A4, respectively (Figure 3H and Supplementary Figure S3H).

GC architecture around exon-intron borders was previously studied (64). It was shown that in low GC content environments introns have undergone lengthening during evolution; hence, the splicing machinery probably recognizes the exon rather than the intron. In high GC content regions introns are relatively short, and therefore introns are probably the units recognized by the spliceosome (64). Therefore, we determined lengths of the flanking introns of each exon in each group (D1 *n* = 3924, D2 *n* = 4806, D3 *n* = 4809, D4 *n* = 4806, D5 *n* = 10 334; A1 *n* = 2978, A2 *n* = 3378, A3 *n* = 3447, A4 *n* = 3327, A5 *n* = 7259). Consistently with the differences in GC levels, groups D1–D4 and A1-A4 possessed longer introns than those in groups D5 and A5, respectively (Figure 3I and Supplementary Figure S3I, **P < 0.01). We hypothesize that in low GC content environments, nucleosomes that mark exons are fixed in their position due to higher levels of GC content in these exons compared to their flanking introns (64). Hence, H1.5 binding is optimally positioned to influence splicing as well. In the low GC content environments, intron lengths are generally longer than in high GC content regions (64). Our data suggest that nucleosome position determines the distance between H1.5 and a splice site, and this distance influences splice site recognition.

### Reduction in H1.5 levels affects inclusion of short exons with relatively long introns

**Figure 4.**
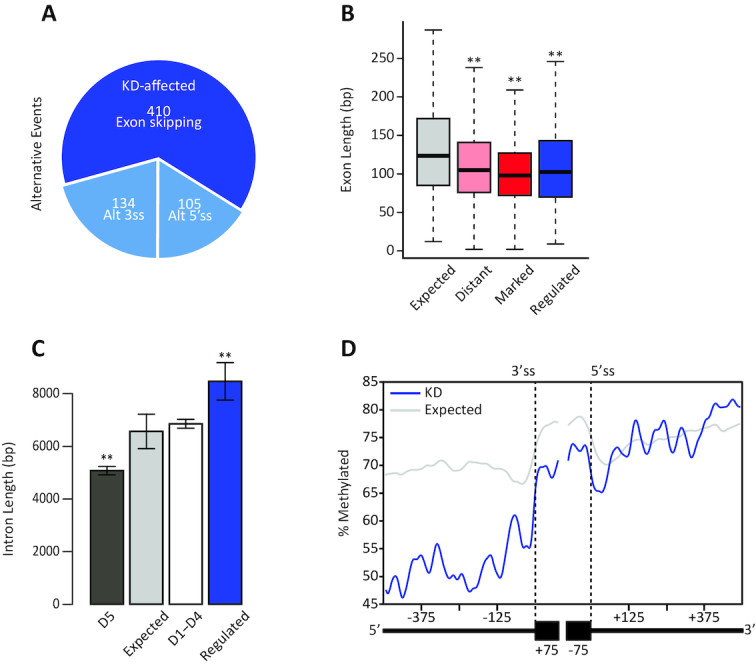
Reduction in H1.5 levels affects inclusion of short exons with relatively long introns. (**A**) Classification and quantification of the changes in alternative splicing in response to H1.5 deficiency: exon skipping, alternative 5′ss selection (Alt 5′ss), and alternative 3′ss selection (Alt 3′ss). Intron retention events were not tested. (**B**) Distribution of the lengths of exons with altered inclusion (blue; *n* = 410), randomly selected exons (orange; *n* = 410), H1.5-distant (pink (*n* = 32 094)), and H1.5-marked (red *n* = 24 711); *P* < 0.01, Wilcoxon rank sum test. (**C**) Mean length of introns flanking the exons with altered inclusion (blue; *n* = 816), randomly selected introns (orange; *n* = 816), exons in D5, and exons in D1–D4; *P* < 0.01, Wilcoxon rank sum test. (**D**) Mean percentage of methylated CpG around exons with altered inclusion in H1.5-deficient cells and a random group (*n* = 10 000) at single-base resolution across 575-bp windows at both junctions.

In order to directly test the effect of linker histone H1.5 on alternative splicing we analyzed previously published RNA-seq datasets from wild-type IMR90 cells and IMR90 cells treated with an siRNA designed to reduce H1.5 levels (45). Using a 10% threshold in differential inclusion level between wild-type and H1.5-deficient conditions, we detected 649 cases of differential inclusion, indicating a possible regulatory role for linker histone H1.5 in alternative splicing. We detected 410 exons (from 392 genes) with differential inclusion between the conditions, 134 cases of differential use of an alternative 3′ss, and 105 cases of differential use of an alternative 5′ss (Figure 4A). To rule out other potential effectors of the observed splicing changes, we compared the expression of the 392 genes that were differentially spliced due to H1.5 reduction in control and H1.5-deficient conditions (Supplementary Figure S4A; left panel) and found a strong correlation (*r* = 0.94, Pearson's product-moment correlation). In addition, we also identified 53 genes that were differentially expressed between control and condition and intersected them with the 392 genes that had splicing changes (Supplementary Figure S4A; right panel). Only two genes intersected. We repeat these steps with a set of 65 splicing factors. The expression of the splicing factors strongly correlated in control and H1.5-deficient conditions (*r* = 0.98, Pearson's product-moment correlation) (Supplementary Figure S4B; left panel) and did not intersect with any of the 53 genes that changed expression (Supplementary Figure S4B; right panel). Hence, the splicing changes we observed were not due to changes in gene expression or changes in splicing factor abundance.

Next, we characterized the 410 exons with altered inclusion rates in the absence of H1.5. We measured the lengths of the exons and compared these to the lengths of H1.5-marked and H1.5-distant exons. To test for statistical significance, we compared these groups to randomly selected internal exons (*n* = 410). In comparison to the mean length of the random sample, exons that were affected by H1.5 reduction were significantly shorter (Figure 4B), similar to the length of H1.5-marked exons. We applied the same approach to introns flanking these exons (*n* = 816), and we found that introns flanking exons affected by H1.5 reduction were significantly longer than those in the randomly selected set (Figure 4C). We also measured the mean proportion of methylated CpG in wild-type IMR90 cells around the regulated exons and compared it to the proportions around a set of 10 000 random exons (Figure 4D). The proportion of methylated CpGs in the upstream intron of the regulated exons was lower than control, with the values been similar to those observed for H1.5-marked exons that were included at low levels (Supplementary Figure S2D). These results suggest that short exons with relatively long introns are sensitive to H1.5 reduction.

### H1.5-marked exons are down regulated in H1.5 deficient IMR90 cells

**Figure 5.**
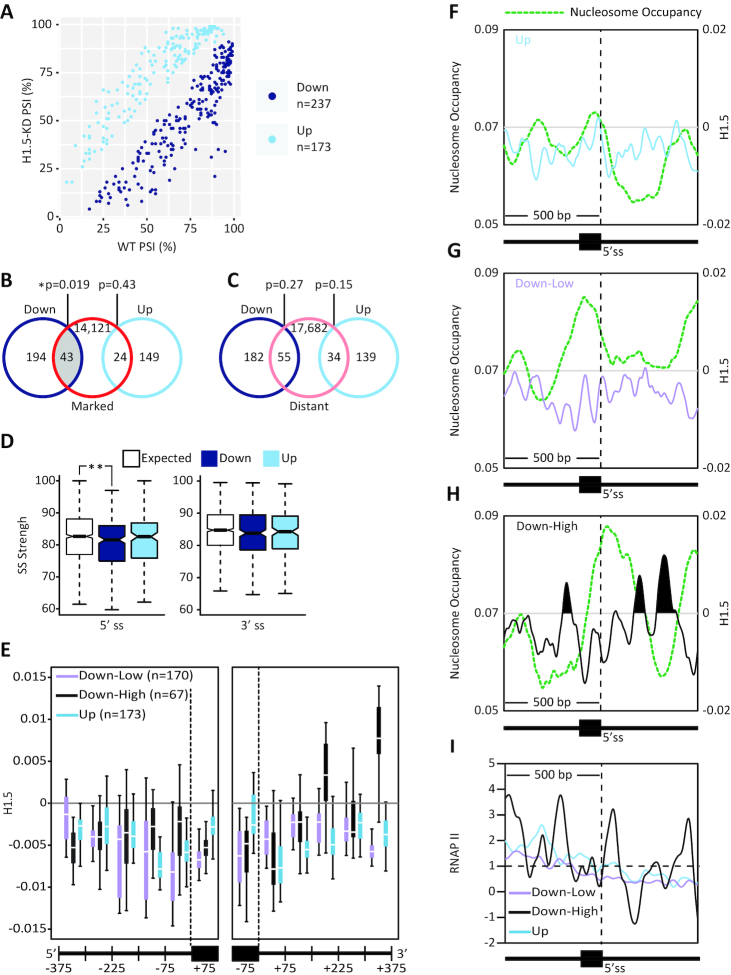
H1.5-marked exons are down regulated in H1.5 deficient IMR90 cells (**A**). The inclusion levels of differential exons in wild-type and H1.5-deficient cells. (**B**) Venn diagram of the intersection between exons with altered inclusion in H1.5-deficient cells, divided into down-regulated and up-regulated, and H1.5-marked exons (red); *P* < 0.05, hypergeometric test. (**C**) Venn diagram of the intersection between exons with altered inclusion in H1.5-deficient cells, divided into down-regulated and up-regulated, and H1.5-distant exons (pink); hypergeometric test. (**D**) 5′ss and 3′ss strength distributions for the down- and up-regulated exons and for a random group of exons (E, white; *n* = 200); *P* < 0.01, Wilcoxon rank sum test. (**E**) Mean H1.5 occupancies at single-base resolution for exons with marginally down-regulated exons (down-low, ΔPSI between −0.25 and 0, purple, *n* = 170), highly down-regulated exons (down-high, ΔPSI < −0.25, black, *n* = 67), and up-regulated exons (up, ΔPSI > 0, cyan, *n* = 173) grouped into bins of 75 bp across 375 bp of intron and 75 bp around the exons at both junctions. (**F**–**H**) Nucleosome and H1.5 signal are displayed together for (F) up-regulated exons, (G) marginally down-regulated exons and (F) highly down-regulated exons. (**I**) RNAP II occupancy is shown for up-regulated exons, marginally down-regulated exons and highly down-regulated exons.

Of the 410 H1.5-influenced exons ∼37% more exons were down-regulated than up-regulated (Figure 5A). Among the 273 exons that were down-regulated, 104 exons were constitutively included in wild-type cells (Supplementary Table S1). Therefore it may be that H1.5 plays a role in constitutive splicing, although here we detected its effects by analysis of alternative splicing. Importantly, only the down-regulated exons intersected significantly with our previously identified H1.5-marked exons (hypergeometric test; *P* < 0.05; Figure 5B). The up-regulated exons displayed no significant intersection (*P* = 0.43). In addition, neither the down-regulated nor the up-regulated exons intersected significantly with H1.5-distant exons (Figure 5C).

We also compared the 5′ss and 3′ss strengths of the down-regulated and the up-regulated exons to the mean splice site strength of randomly selected exons (*n* = 200) (Figure 5D). The down-regulated exons had weaker 5′ss than the randomly selected exons, whereas the up-regulated exons had no such difference, suggesting that H1.5 is involved in 5′ss recognition.

Next, we further divided the regulated exons according to the magnitude of the change in inclusion level (ΔPSI) between control and H1.5-deficient conditions (down-low: ΔPSI ≥ −0.25; down-high: ΔPSI < −0.25). We plotted the H1.5 occupancies grouped into six bins of 75 bp each around the 3′ss and the 5′ss. Around the weak 5′ss, the highly down-regulated group exhibited a high H1.5 signal (Figure 5E). These results suggest that H1.5 aids the 5′ss recognition of the sensitive exons. We then plotted the nucleosome and H1.5 occupancies for each group around the 5′ss (Figure 5F–H). Interestingly we found that only down-regulated exons were occupied by a nucleosome and only the highly down-regulated exons were also bound by H1.5. This result suggests that exons on nucleosomes are more sensitive to chromatin changes, with higher sensitivity for those that are also marked by H1.5, than are unmarked exons. Further, we measured RNAP II signal in all three groups and found that RNAP II stalls near nucleosomes with H1.5 (Figure 5I), suggesting that these combined chromatin features affect RNAP II progression over a nucleosome.

We also evaluated the genetic function of the genes that had down-regulated exons and the genes that had up-regulated exons when H1.5 was depleted. We found that genes with down-regulated exons were more significantly enriched for the same terms as H1.5-marked genes than were the genes with up-regulated exons (Supplementary Figure S5A and B). These terms were alternative splicing, nucleotide-binding, metal-binding, and beta-transducin (WD) repeat.

### The upstream introns of highly down-regulated exons possess a regulatory region.

**Figure 6.**
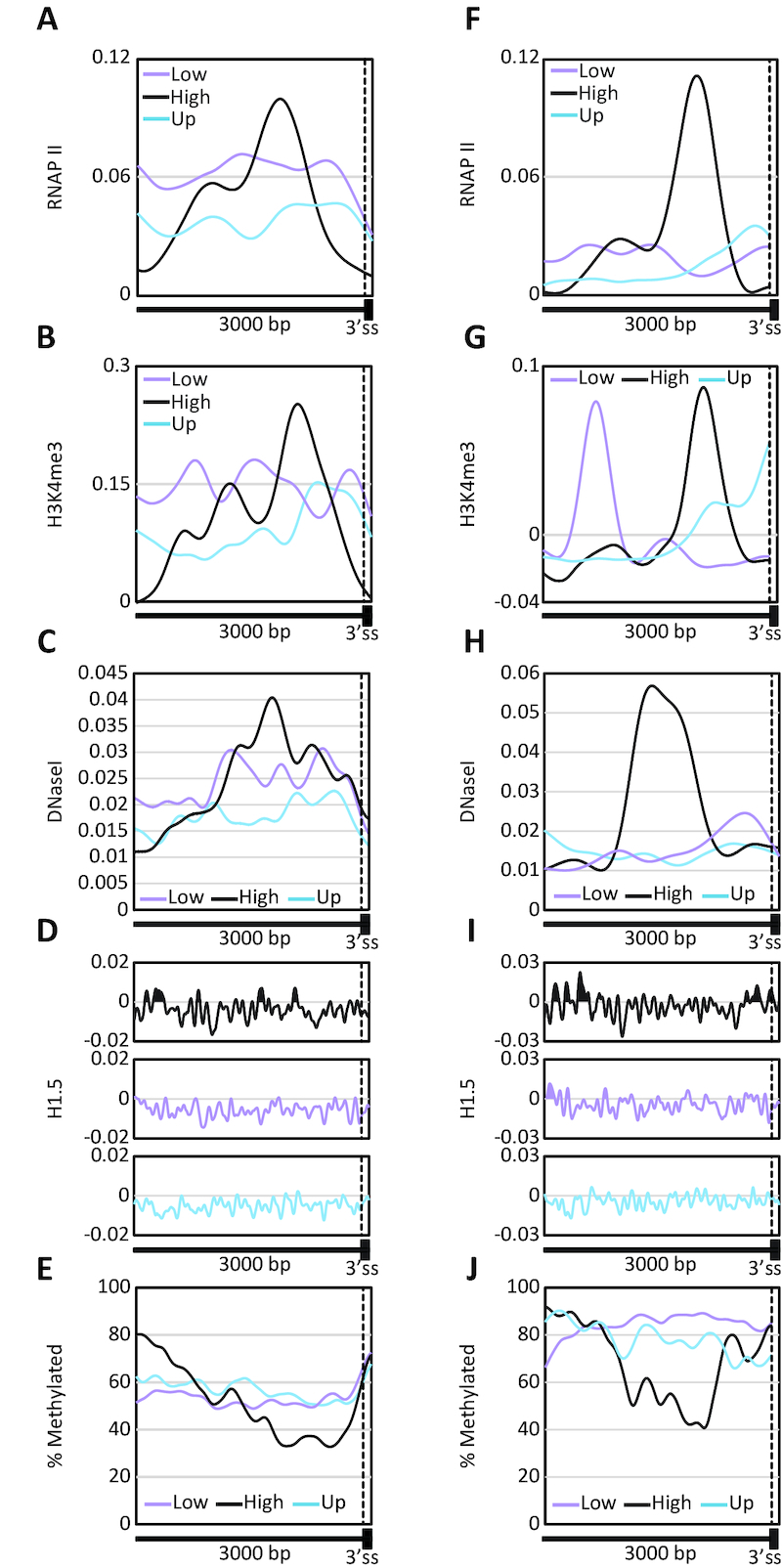
The upstream introns of highly down-regulated exons possess a regulatory region. (**A**) Mean RNAP II occupancy at single-base resolution across 3,000 bp of intron and 100 bp up-regulated exons (up), marginally down-regulated exons (down-low), and highly down-regulated exons (down-high). (**B**) Mean H3K4me3 enrichment at single-base resolution across 3000 bp of intron and 100 bp up-regulated exons, marginally down-regulated exons, and highly down-regulated exons. (**C**) Mean DNaseI enrichment at single-base resolution across 3000 bp of intron and 100 bp up-regulated exons, marginally down-regulated exons, and highly down-regulated exons. (**D**) Mean H1.5 occupancy at single-base resolution across 3000 bp of intron and 100 bp up-regulated exons, marginally down-regulated exons, and highly down-regulated exons. (**E**) Mean proportions of CpG methylation at single-base resolution across 3000 bp of intron and 100 bp up-regulated exons, marginally down-regulated exons, and highly down-regulated exons. (**F–J**) The same as panels A–E, only the analysis excluded the first, second, third, and fourth exon in each gene.

We observed RNAP II accumulation in IMR90 cells approximately 1,000 bp upstream of the exons that were highly down-regulated by H1.5 reduction (Figure 6A). These regions were also enriched for H3K4me3 (Figure 6B), DNase I hypersensitive sites (Figure 6C), and H1.5 (Figure 6D) and were significantly depleted of DNA CpG methylation (Figure 6E). Since RNAP II pausing, CpG methylation, and H3K4me3 are all features found near promoters, we repeated the analysis of these markers limiting the analysis only to exons located at positions fifth or higher in their gene. This analysis yielded the same results (Figure 6F–J) ruling out the possibility that the decrease in exons’ inclusion we observed in H1.5-deficient cells, is due to alternative promoter activation. It is known that H3K4me3 and DNA CpG methylation are mutually exclusive (65), and H3K4me3 can co-localize with RNAP II (66). It might be that H3K4me3 prevents CpG methylation and promotes RNAP II slowing which enhances exons’ inclusion.

### H1.5 depletion releases RNAP II pausing and leads to exon skipping

**Figure 7.**
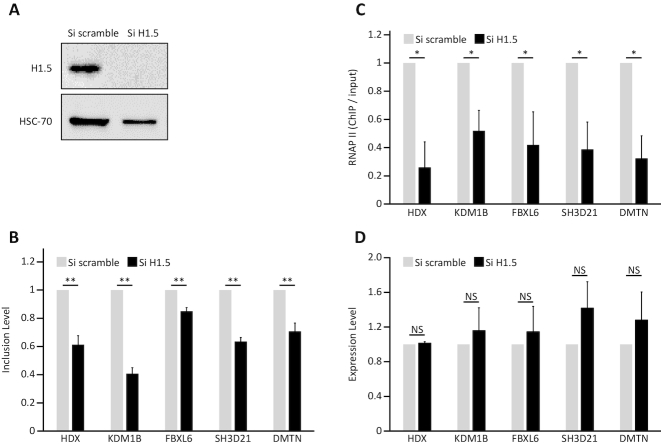
H1.5 depletion releases RNAP II pausing and leads to exon skipping. (**A**) H1.5 expression in IMR90 cells was reduced using siRNA targeting *H1.5*. Control cells were treated with a non-targeted siRNA. At 48 h after transfection, proteins were extracted and examined by western blotting using H1.5 and HSC-70 antibodies. (**B**) At 48 h after transfection with siRNA targeting *H1.5* or control siRNA, total RNA was extracted, and qRT-PCR was used to analyze inclusion of alternatively spliced exons from the indicated genes. Inclusion is relative to levels in cells treated with control siRNA. Plotted are means of three independent experiments ± SEM. (**C**) At 48 h after transfection, ChIP-qPCR assay was performed using total RNAPII antibody. Pausing of RNAPII was analyzed in the regions of the indicated exons using primers at the end of an intron adjacent to an exon. Plotted is mean ChiP signal (± SEM) relative to input normalized to signal in cells treated with control siRNA from three independent experiments. (**D**) At 48 h after transfection, total RNA was extracted following relative qRT-PCR analysis. Expression mRNA levels of the five genes were measured in three independent experiments and normalized to *GAPDH* gene. Plotted are means of three independent experiments ± SEM.

Finally we aimed to validate our findings. We depleted IMR90 cells of H1.5 using an siRNA as described previously (45). After 48 hours, the quantity of H1.5 protein in treated cells was ∼50% of that in control cells (Figure 7A). Using qRT-PCR, we evaluated five H1.5-marked exons that were highly down-regulated in response to H1.5 reduction. We chose exons of 50–120 bp in length with more than 50% inclusion level in wild-type cells, and with no significant change in their host gene expression upon H1.5 reduction (delta FPKM < 1). There was a significant decrease in inclusion level of these exons in IMR90 cells depleted of H1.5 (Figure 7B). This supports the validity of our genome-wide analysis. In addition, we measured RNAP II levels near the upstream intron-exon borders of these exons using a ChIP assay (Figure 7C). We found that in these exons RNAP II levels were reduced in H1.5-deficient cells compared to control cells, implying that lack of H1.5 enables rapid RNAP II elongation and leads to exon skipping. To ensure that these findings were not due to a decrease in genes expression, we measured mRNA level of the five selected genes in control and H1.5-deficient cells (Figure 7D). No significant change in genes expression was detected, indicating that the reduction in exons inclusion and RNAP II levels was caused by H1.5 absence from the tested H1.5-marked exons.

## DISCUSSION

Here, we studied the role of histone H1.5 in marking splice sites. We found that H1.5 binds over splice sites of short exons: 75% of H1.5-marked exons were 127 bp or shorter. This suggests that these exons are biased toward maintaining a length no longer than the length of the DNA surrounding nucleosomes. The length of an exon that is encoded by DNA bound to a nucleosome dictates the position of the splice sites relative to H1.5. We found that the distance between H1.5 and a splice site can have an effect on the exon inclusion level in the mature mRNA.

RNAP II passage through a nucleosome involves the disassociation of DNA–histone interactions, resulting in either the removal of a single H2A/H2B dimer or the entire nucleosome core (67,68). The ability of RNAP II to overcome the nucleosome barrier improves with the formation of the elongation complex. The elongation complex associates with the H2A/H2B dimer (69) and with linker histone variant H1.0 (34). Other studies demonstrated that H1x associates with RNAP II in actively transcribed regions (32) and that H1.2 can interact with serine 2 phosphorylated form of RNAPII and with PAF1 elongation complex (33). Linker histones are located near the nucleosome dyad. Therefore, it is possible that H1.5 interacts with RNAP II directly or interferes with the removal of the H2A/H2B dimer. The hypothesis that RNAP II pausing is the result of direct binding with H1.5 is strengthen by our observation of a more dominant effect on splicing when H1.5 occupied the 5′ss than when it was bound to the 3′ss. Since RNAP II transcribes the 3′ss prior to the 5′ss, H1.5 bound to a 3′ss must dissociate before transcription of either splice site, limiting its ability to influence RNAP II pausing. In contrast, H1.5 can remain bound to the 5′ss through transcription of both the splice sites. Hence, H1.5 bound to the 5′ss has a better potential to regulate splicing. Interestingly, the RNAP II tail associates with U1 snRNP, which binds the 5′ss on the pre-mRNA (25). Along this line it is important to note that down-regulated exons in H1.5 deficient cells displayed a weak 5′ss occupied by H1.5, and that overall we found roughly 37% more cases of H1.5 occupying the 5′ss than 3′ss.

The presence of CpG sites around H1.5-marked and H1.5-distant exons correlates with H1.5 binding. We speculate that methylation enhances H1.5 binding; however, it may be that H1.5 binding promotes DNA methylation, as some linker histones were shown to bind certain DNA methyltransferases (61). In support of the former hypothesis, we found that H1.5-distant exons exhibit characteristics between H1.5-marked exon and unmarked exons. We suggest that high levels of methylation promote H1.5 binding over an area. H1.5-distant exons had a lower nucleosome occupancy with a smaller difference between the exon and intron areas compared to H1.5-marked exons (Figure 2H). Therefore, H1.5 do not bind near the splice sites of H1.5-distant exons. Hence, H1.5 binding distantly does not promote RNAP II pausing, and we did not observe an effect on the inclusion of these exons when H1.5 was down-regulated.

Finally, considering that H1.5 binds thousands of exons, it could be argued that H1.5 reduction should have resulted in many more cases of decreased inclusion than observed. It is likely that for many of the constitutive exons that associate with H1.5, additional signals facilitate constitutive selection by the splicing machinery even in the absence of H1.5, and thus reduction in H1.5 level has no apparent effect on their inclusion (70). Moreover, several studies have shown that deficiencies in linker histones are compensated by other linker histone subtypes (71). It might be that other linker histones bind the locations H1.5 would bind, if present, and promote RNAP II pausing or other functions that facilitate splicing. For instance, linker histone H1.0 associates with components of U2 (SF3A and SF3B) and U5 snRNPs and with splicing factors including U2AF, SRSF1, and SRSF2, which are also part of the basal splicing machinery and the regulatory system (34). It is also possible that changes in linker histones variant expression lead to the decrease in RNAP II pausing in H1.5 deficiency cells. To address the exact mechanism by which linker histones affect splicing, further investigation is needed.

## Supplementary Material

gkz338_Supplemental_File
