# Supplementary material for: Histone H1.5 binds over splice sites in chromatin and regulates alternative splicing
Source: Nucleic Acids Res. 2019 May 11;47(12):6145–59. doi: 10.1093/nar/gkz338 (PMC6614845; doi:10.1093/nar/gkz338)
Supplement: gkz338_Supplemental_File [file gkz338_supplemental_file.pdf]

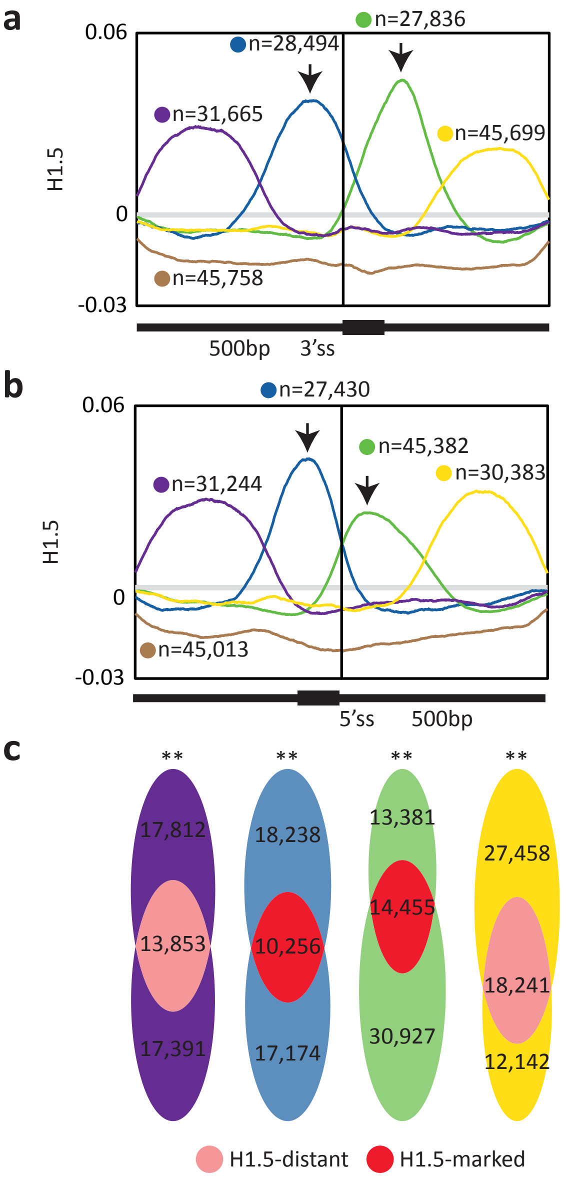
a
0.06
0
-0.03
H1.5
n=27,836
n=28,494
n=31,665
n=45,699
n=45,758
500bp
3’ss
b
0.06
0
-0.03
H1.5
n=27,430
n=45,382
n=30,383
n=31,244
n=45,013
5’ss
500bp
c
**
**
**
**
17,812
13,853
17,391
18,238
10,256
17,174
13,381
14,455
30,927
27,458
18,241
12,142
H1.5-distant
H1.5-marked

Figure S1

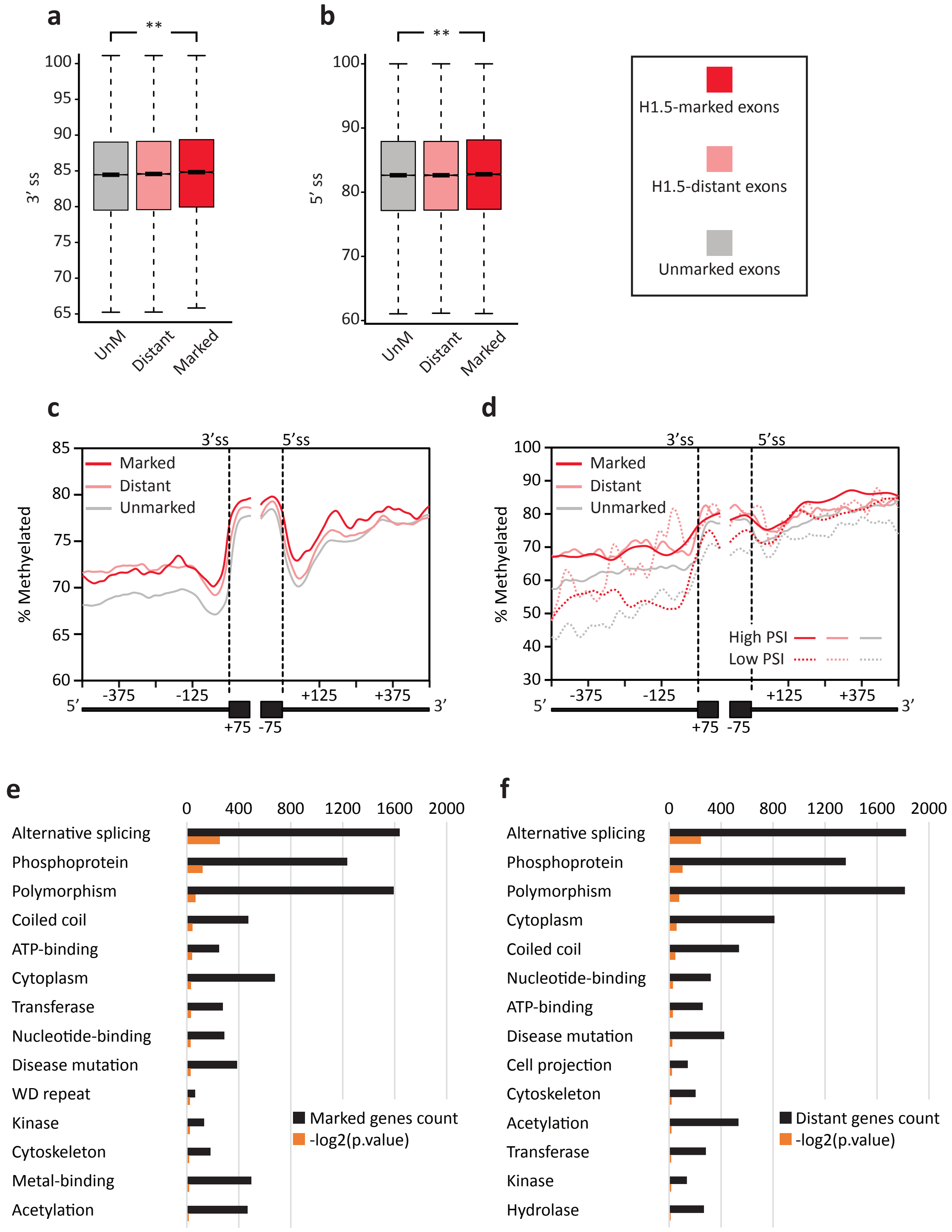
a
b
**
3' ss
5' ss
UnM
Distant
Marked
H1.5-marked exons
H1.5-distant exons
Unmarked exons
c
d
3'ss
5'ss
Marked
Distant
Unmarked
% Methyelated
-375
-125
+125
+375
5'
3'
+75
-75
High PSI
Low PSI
e
f
Alternative splicing
Phosphoprotein
Polymorphism
Coiled coil
ATP-binding
Cytoplasm
Transferase
Nucleotide-binding
Disease mutation
WD repeat
Kinase
Cytoskeleton
Metal-binding
Acetylation
Marked genes count
-log2(p.value)
Cell projection
Hydrolase
Distant genes count

Figure S2

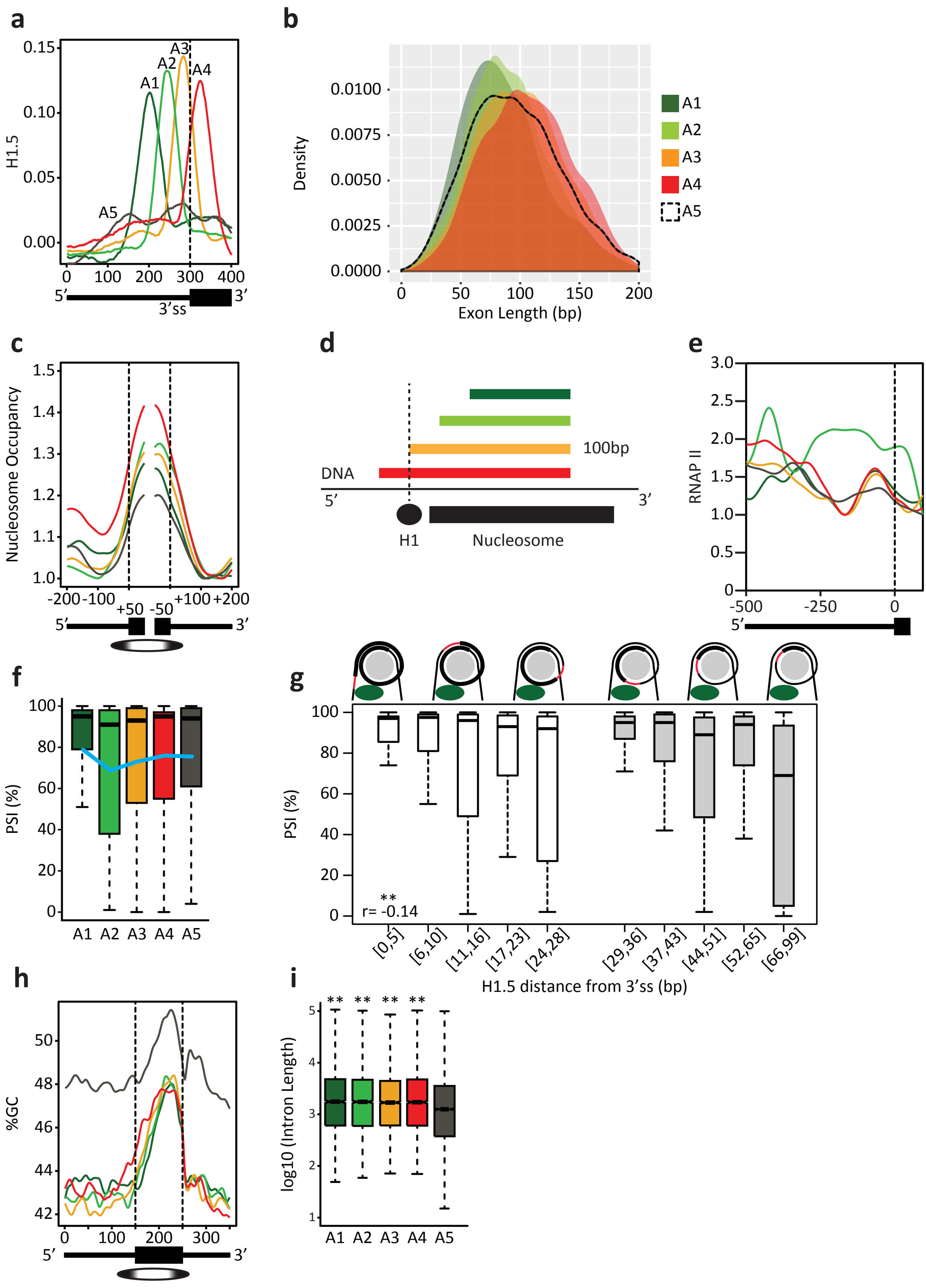

a
H1.5
0.15
0.10
0.05
0.00
A1
A2
A3
A4
A5
0
100
200
300
400
5′
3′
3′ss
b
Density
0.0100
0.0075
0.0050
0.0025
0.0000
0
50
100
150
200
Exon Length (bp)
A1
A2
A3
A4
A5
c
Nucleosome Occupancy
1.5
1.4
1.3
1.2
1.1
1.0
-200
-100
+50
-50
+100
+200
5′
3′
d
100bp
DNA
5′
3′
H1
Nucleosome
e
RNAP II
3.0
2.5
2.0
1.5
1.0
0.5
0
-500
-250
0
5′
f
PSI (%)
100
80
60
40
20
0
A1
A2
A3
A4
A5
g
PSI (%)
100
80
60
40
20
0
**
r= -0.14
[0,5]
[6,10]
[11,16]
[17,23]
[24,28]
[29,36]
[37,43]
[44,51]
[52,65]
[66,99]
H1.5 distance from 3′ss (bp)
h
%GC
50
48
46
44
42
0
100
200
300
5′
3′
i
log10 (Intron Length)
5
4
3
2
1
** ** ** **
A1
A2
A3
A4
A5

Figure S3

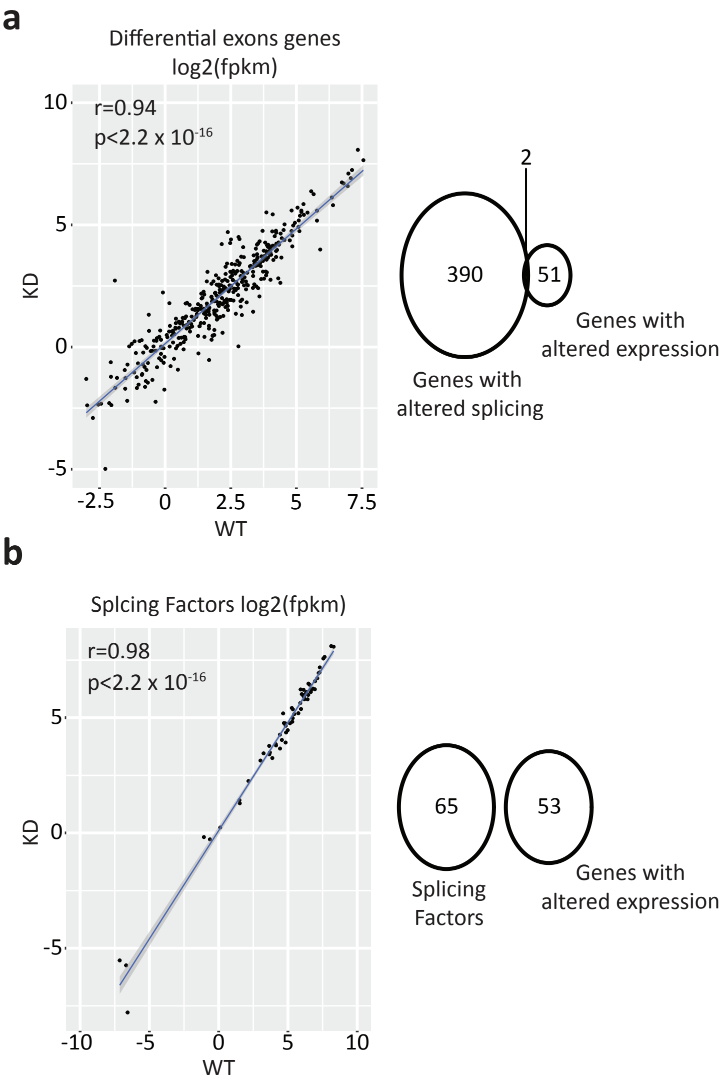
a
Differential exons genes
log2(fpkm)
r=0.94
p<2.2 x 10-16
10
5
0
-5
KD
-2.5
0
2.5
5
7.5
WT
2
390
51
Genes with
altered expression
Genes with
altered splicing
b
Splcing Factors log2(fpkm)
r=0.98
p<2.2 x 10-16
5
0
-5
KD
-10
-5
0
5
10
WT
65
53
Splicing
Factors
Genes with
altered expression

Figure S4

a

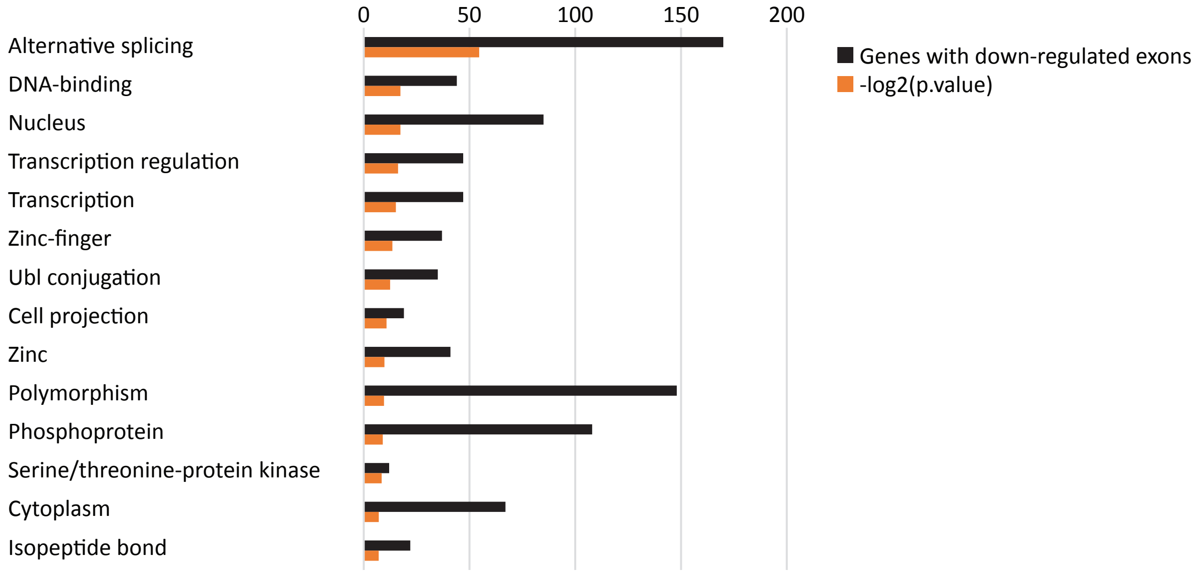
0
50
100
150
200
Alternative splicing
DNA-binding
Nucleus
Transcription regulation
Transcription
Zinc-finger
Ubl conjugation
Cell projection
Zinc
Polymorphism
Phosphoprotein
Serine/threonine-protein kinase
Cytoplasm
Isopeptide bond
Genes with down-regulated exons
-log2(p.value)

b

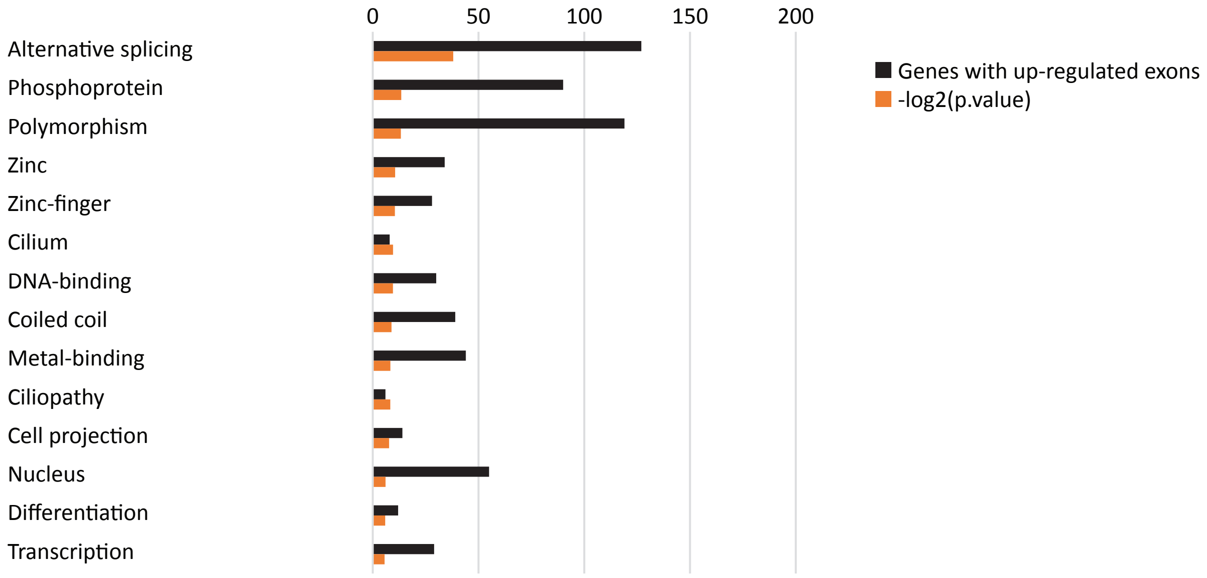
0
50
100
150
200
Alternative splicing
Phosphoprotein
Polymorphism
Zinc
Zinc-finger
Cilium
DNA-binding
Coiled coil
Metal-binding
Ciliopathy
Cell projection
Nucleus
Differentiation
Transcription
Genes with up-regulated exons
-log2(p.value)

Figure S5

Table S1. Counts of the exons affected by H1.5 knockdown, divided by their type, in both wildtype and knockdown conditions

Condition	Type	Up	Down
Wildtype	Consitutive	0	104
	Alternative	173	133
Knockdown	Consitutive	79	0
	Alternative	94	237

SUPPLEMENTARY FIGURE LEGENDS

Figure S1. Clustering H1.5 occupancy into five groups. (**A** and **B**) H1.5 occupancy at single-base resolution near (A) 3'ss and (B) 5'ss for five groups identified using *k*-means clustering (indicated by color). Group sizes are indicated. (**C**) Venn diagrams of numbers of exons in two clusters that has elevated signal upstream to the 3'ss (purple), two clusters that has elevated signal over the 3'ss (blue), two clusters that has elevated signal over the 5'ss (green), and two clusters that has elevated signal downstream to the 5'ss (yellow); $p < 2.2\times10^{-16}$, hypergeometric test.

Figure S2. H1.5-marked exons have strong splice sites and are enriched in DNA CpG methylation. (**A** and **B**) H1.5-marked exons have strong 3'ss and 5'ss sequences. The splice site strength distributions of H1.5-marked, H1.5-distant, and H1.5-unmarked exons are shown; $p < 0.01$, Wilcoxon rank sum test. (**C**) Mean percentage of methylated CpG sites at single-base resolution across the exon-intron junctions of H1.5-marked, H1.5-distant, and H1.5-unmarked exons. Both the 3'ss and the 5'ss sites are shown, with 500 bp of intron sequences and 75 bp of exon sequences. (**D**) Mean percentage of methylated CpG sites at single-base resolution across the exon-intron junctions. Signal is shown for alternatively spliced H1.5-marked exons with PSI < 30% (n=185), and with PSI > 70% (n=752), for alternatively spliced H1.5-distant exons with PSI < 30% (n=250), and with PSI > 70% (n=1,065), and for unmarked alternatively spliced exons with PSI < 30% (n=1,239), and with PSI > 70% (n=5,831). (**E**) Significantly enriched biological process GO terms in genes with H1.5-marked exons. (**F**) Significantly enriched biological process GO terms in genes with H1.5-distant exons.

Figure S3. H1.5 distance from a splice site affects exon inclusion. (**A**) The exons marked by H1.5 at their 5'ss (n=14,455) were aligned at 5'ss junction and clustered into five groups (A1-A5) based on H1.5 mean occupancies across 100 bp of exon sequence and 300 bp of intron sequence at single-base resolution. (**B**) Exon length (bp) distribution for each of the five groups A1-A5; (**C**) Mean nucleosome occupancy at single-base resolution for each of the groups A1-A5 across 200 bp of intron and 50 bp of exon. Signals were normalized to the lowest value for each individual cluster. Ellipse denotes the likely nucleosome location. (**D**) Drawing of the absolute distance between the 3'ss and H1.5. (**E**) Mean RNAP II occupancy for each of the five groups A1-A5. The signals were normalized to the lowest value of each signal. (**F**) Percentage of alternative exon inclusion within each group A1-A5. (**G**) Percentage of alternative exon inclusion relative to the absolute distance of H1.5 from a splice site. Each box presents a different distance. The drawing shows the distance between H1 (green) and the splice site (red); $p < 0.01$, $r = -0.14$, Pearson's product-moment correlation (**H**) Mean GC content at single-base resolution across a 350-bp region around the exons in groups A1-A5 with the exons aligned at end points. Ellipse marks the likely

nucleosome location. (**I**) Flanking intron length (bp) distribution for each of the groups A1-A5 **$p < 0.01$, Wilcoxon rank sum test.

Figure S4. Changes in splicing due to H1.5 reduction are not the results of differential gene expression. (**A**) Left panel: Plot of gene expression (in log2 fpkm) of the genes with splicing altered due to H1.5 reduction in control and H1.5-deficient conditions. The regression line is plotted (blue), and the correlation and p value of Pearson's product-moment correlation is given. Right panel: Intersection between genes with altered expression and genes with exons sensitive to H1.5 reduction. (**B**) Left panel: Plot of gene expression (in log2 fpkm) of splicing factors in control and H1.5-deficient conditions. Right panel: Intersection between genes with altered expression and genes encoding splicing factors.

Figure S5. Genes with exons sensitive to H1.5 depletion have the same functions as genes with H1.5-marked exons. (**A**) Significantly enriched biological process GO terms in genes containing down-regulated exons. (**B**) Significantly enriched biological process GO terms in genes with up-regulated exons.

Table S1

H1.5 deficiency affects the selection of both constitutively and alternatively spliced exons.
